# A Case of Osteomyelitis after Calcaneal Fracture Treated by Antibiotic-Containing Calcium Phosphate Cements

**DOI:** 10.1155/2018/9321830

**Published:** 2018-06-12

**Authors:** Yoohak Kim, Fumiaki Inori, Kiyotaka Yamanaka, Shouichi Murakami, Eri Narita, Kazumasa Yamamura, Hiroyuki Yasuda, Makoto Fukuda, Sadahiko Konishi, Yukihide Minoda

**Affiliations:** ^1^Department of Orthopedic Surgery, JR Osaka Railway Hospital, Osaka, Japan; ^2^Department of Orthopedic Surgery, Osaka City University Graduate School of Medicine, Osaka, Japan

## Abstract

Calcaneal osteomyelitis (CO) is considered to be difficult to cure when it turned into a chronic phase. We report one case of calcaneal osteomyelitis which arises after the operation of calcaneal fracture. Remission was obtained by performing curettage of the infected cancellous bone of the calcaneal body and filling antibiotic-containing calcium phosphate cements (CPC) within its bone defect. This one-stage surgery is useful to treat calcaneal osteomyelitis.

## 1. Introduction

It was reported that calcaneal osteomyelitis (CO) accounted for 7%-8% of all osteomyelitis cases in adults [1]. In the literature, often, wound infection, soft tissue infection, and bone infection would not be differentiated. Schildhauer et al. quantified the calcaneal rate of infections to 11% [2]. Aseptic necroses of the wound edge especially after extended lateral approaches to the calcaneus were described in the literature between 2 and 27.3% [3–5]. Delayed healing or postoperative infection might occur up to 25% [6–11].

The clinical principal of treatment of this disease is antibiotic administration, irrigation, and debridement [12], whereas when it turned into a chronic phase, procedures of the treatment become difficult. Heier et al. postulated in 2003 that “the extent of soft tissue damage determines the therapeutical result” [13]. In this respect, the early soft tissue coverage plays an important role, so that early diagnosis and appropriate operative treatment are indispensable. The preservation of the calcaneus and thus a functional pedal anatomy is the main target during the infect sanitation. This is not always feasible. Depending on the local situation, the spectrum of surgical procedures includes partial calcaneal resection, calcanectomy, and lower leg amputation. According to Lehmann et al. and Bollinger and Thordarson, partial calcanectomy is a decent alternative to lower leg amputation in cases of strictly local infection [14, 15]. The authors mentioned that partial calcaneal resection may be performed if the inflammatory process does involve less than 50% of the heel [16]. In these circumstances, the sufficient hind foot blood supply seems to be the central problem [17, 18]. Syme amputation may also be performed in special cases.

We report one case of calcaneal osteomyelitis arising after the pinning operation, and its remission was obtained by performing curettage of the infected cancellous bone of the calcaneal body and filling antibiotic-containing calcium phosphate cements (CPC).

## 2. Case Report

**Figure 1:**
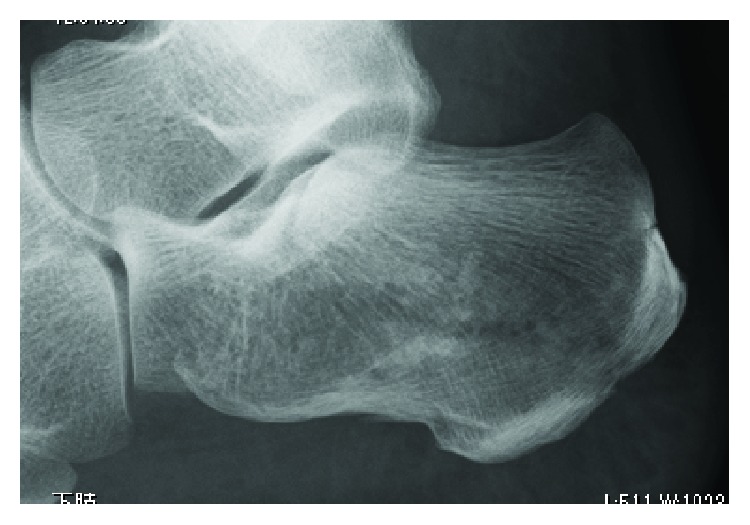
Lateral radiograph of the left foot following injury. X-ray shows the tongue-type fracture of the left calcaneus.

**Figure 2:**
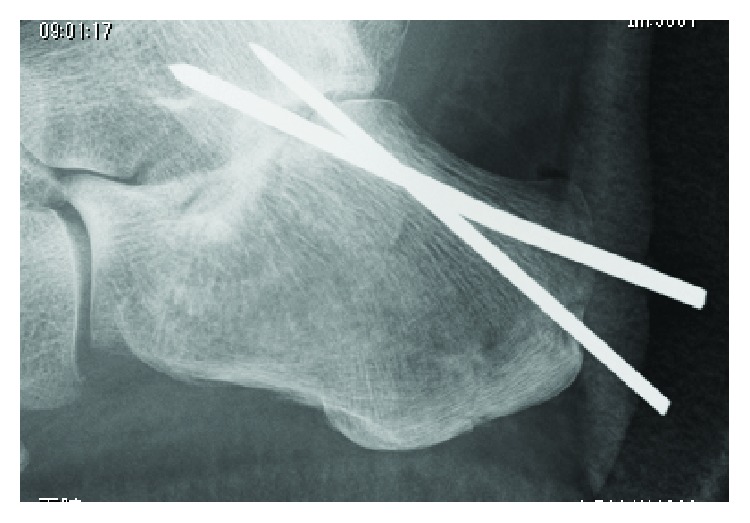
Lateral radiograph of the left foot following the first surgery. X-ray shows that the left calcaneus was almost completely reduced with two Steinmann pins.

A 51-year-old female had an injured left foot by falling down from home stairs. The next day, she was admitted to our hospital and was diagnosed with closed tongue-type calcaneal fracture (Figure 1). Operation was performed using 2 pins of the Steinmann pin by the Westhues method (Figure 2). A fixed cast and 2 pins were removed at the same time on the 37th postoperative day, and there was no potential for infection at that time. Nevertheless, she was admitted to our hospital with a complaint about heel pain and fever exceeding up to 40 degrees centigrade, after 9 days from the pin removal.

**Figure 3:**
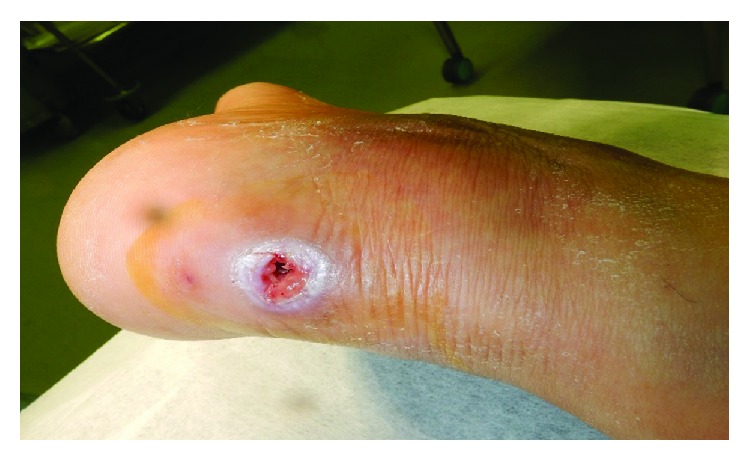
Left heel as seen from the posterior aspect after 9 days from the pin removal. Skin redness, swelling, and pus-like discharge were observed around the surgical site.

**Figure 4:**
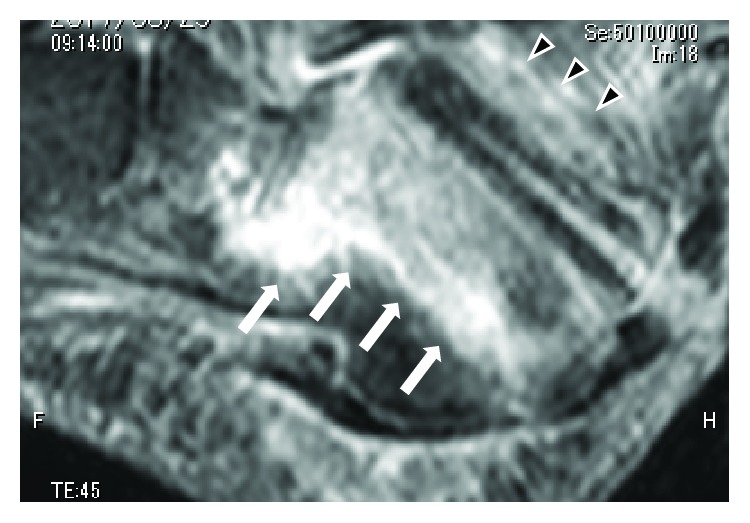
Fat suppressed T2-weighted left ankle magnetic resonance sagittal images before the 2nd operation. High-intensity area was confirmed around the pin tract (black triangle) and body of calcaneus (white arrow). Abscess formation was suspected.

On the examination, skin redness, swelling, and pus-like discharge were observed around the surgical site (Figure 3). Plain X-ray showed hyperpermeability of the calcaneus, and magnetic resonance images confirmed a diagnosis of osteomyelitis of the calcaneus as well as an abscess formation (Figure 4). White blood cell count (WBC: 9.9 × 10^3^/*μ*l) and C-reactive protein (CRP: 10.06 mg/dl) were elevated. And methicillin-sensitive *Staphylococcus aureus* (MSSA) was cultured from the discharge.

**Figure 5:**
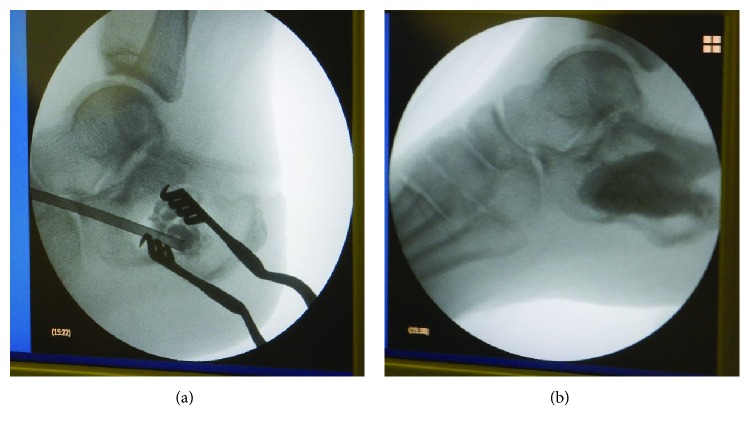
Intraoperative fluoroscopy of the left calcaneus at the 2nd operation. (a) Vancomycin-containing CPC was poured from the lateral side. (b) Defect of the calcaneal body and pin tracts were filled with CPC. We carefully confirmed that there were no leaks of CPC in the subtalus joint intraoperatively.

**Figure 6:**
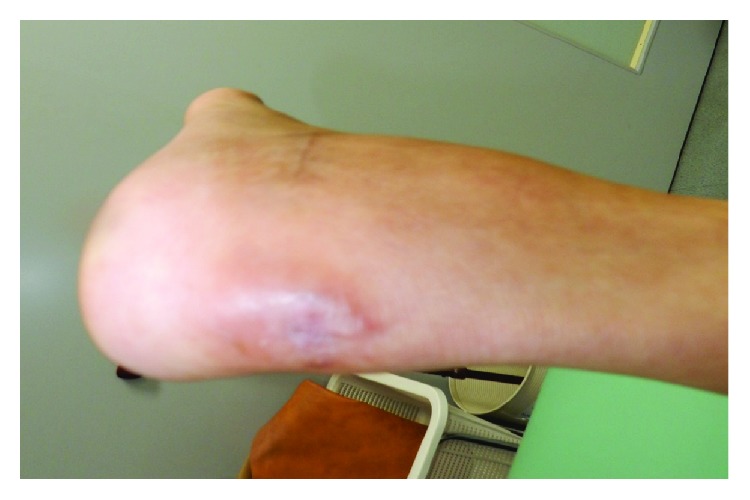
Left heel as seen from the posterior aspect after 6 months from the 2nd operation. The pin tract's fistula was completely closed, and there was no recurrence of infection.

Intravenous antibiotic therapy was administrated immediately (cefazolin 2 g × 3/day), and the next day, the patient underwent irrigation of the surgical site and surgical pus drainage. Fever fell down, and inflammatory aspects disappeared within few days; however, the discharge from the drainage continued on 7 postoperative days. MSSA was cultured again from the discharge, so that we can diagnose whether calcaneal osteomyelitis was not cured completely. 12 days after the 2nd surgery, the patient underwent radical debridement of the calcaneal bone marrow using Ollier's lateral approach and irrigation with natural saline was performed. Subsequently, calcium phosphate cement (CPC) (Hoya Medical, Tokyo, Japan) with vancomycin was implanted at the defected site of the calcaneus (Figure 5). MSSA was also cultured positive from the bone marrow of the calcaneus. Intravenous antibiotic therapy was continued for 7 days, and it was changed to antibiotics per oral (minomycin 200 mg × 2, rifampicin 450 mg × 1) and continued for 30 days. CRP turned negative on the 10th postoperative day, and the pin tract's fistula was completely closed on the 14th postoperative day. Osteomyelitis seemed to be controllable, and 1/3 partial weight bearing was started from 14 postoperative days. Weight bearing was raised every 1 week as 1/2 and 2/3, and full weight bearing had been completed on the 35th postoperative day. On the 6th postoperative month, fistula was completely closed and there was no recurrence of infection (Figure 6). The patient could walk normally without a cane, and we considered that complete remission of osteomyelitis was obtained.

## 3. Discussion

To treat CO, wide surgical debridement, skeletal stabilization, and administration of antibiotics are the main steps to eradicate sepsis [19, 20]. However, the reconstruction of the resulting skeletal and soft tissue defects is often complex. In contrast to the more proximal segments of the leg, the availability of soft tissue for the coverage of full-thickness defects with local or regional flaps is limited [21, 22]. Reconstruction of skeletal defects can be accomplished with bone grafting [23]. However, large defects require complex reconstructive procedures, such as distraction osteogenesis, vascularized bone grafting, or transfer of free flaps [19, 24, 25]. Finally, toe or ray amputations and more extensive amputation procedures in cases of diffuse osteomyelitis can be a limb- and life-saving procedure in a certain group of frail patients [19].

To overcome these large bone defect problems, a cement spacer was considered a good candidate. Polymethyl methacrylate (PMMA) had been the first alternative as a bone filling cement spacer for bone defect. However, it did not have biodegradability; hence, 2nd-stage surgery such as removal and cancellous bone graft was demanded after the 1st-stage surgery [26]. CPC was developed as a bone filling cement spacer and used mainly in bone defects caused by bone tumor or bone fracture because CPC has the tendency to change its form easily to fix the filling part. Recently, the treatment method of osteomyelitis using antibiotic-containing CPC had started to be reported [27].

In this case, we selected 1-stage surgery using antibiotic-containing CPC because of the reasons stated below: the operation was performed within 2 weeks after the diagnosis of osteomyelitis, the patient was healthy without complications such as diabetes, and the detected organism was MSSA which had sensitivity for several antibiotics. With regard to this kind of 1-stage surgery technique, Nan et al. identified that calcium sulfate cement could induce the formation of the membrane in the same way as PMMA and suggested the possibility of 1-stage surgery owing to its degradability [28]. However, they finally removed calcium sulfate and reconstructed bone defect with cancellous bone graft for several reasons; our case accomplished 1-stage surgery with antibiotic-containing CPC.

Recently, investigations addressing the similarities and differences between PMMA and CPC as two types of cement spacer have drawn wide attention. CPC have proved to be a viable carrier of local antimicrobial agent allowing the prolonged release of gentamicin sulfate or tobramycin compared to the PMMA [29]. In an *in vitro* study, Yang et al. investigated the biological safety, biomechanics, and tissue compatibility of CPC and PMMA mixed in different ratios and concluded that CPC had excellent biological properties, whereas mechanical properties were inferior to those of PMMA [30].

Taken together, the previous and present studies have confirmed that calcium phosphate cement, as a novel delivery vehicle, possesses similar effectiveness as PMMA, but a clear characteristic of total biodegradability highlights its superiority over PMMA. However, it should be noted that calcium phosphate is less strong than PMMA.

## 4. Conclusion

CPC is very useful as a bone filling agent for bone defects because it could change its form freely. And it is also useful as an agent for the treatment of calcaneal osteomyelitis which failed to preserving treatment.
